# Elastin-derived peptides (EDPs) as a potential pro-malignancy factor in human leukemia cell lines

**DOI:** 10.1007/s12026-024-09511-7

**Published:** 2024-07-05

**Authors:** Konrad A. Szychowski, Bartosz Skóra

**Affiliations:** Department of Biotechnology and Cell Biology, Medical College, University of Information Technology and Management in Rzeszow, Sucharskiego 2, 35-225 Rzeszow, Poland

**Keywords:** Leukemia, Elastin-derived peptides, c-SRC kinase, Differentiation, VGVAPG, VVGPGA

## Abstract

**Abstract:**

The extracellular matrix (ECM) is currently considered to be an important factor influencing the migration and progression of cancer cells. Therefore, the aim of our study was to investigate the mechanism of action of elastin-derived peptides in cancerous cells derived from the immunological system, i.e., HL-60, K562, and MEG-A2 cell lines. Moreover, an attempt to clarify the involvement of c-SRC kinase in EDP mechanism of action was also undertaken. Our data show that the VGVAPG and VVGPGA peptides are not toxic in the studied cell lines. Moreover, due to the involvement of KI67 and PCNA proteins in the cell cycle and proliferation, we can assume that neither peptide stimulates cell proliferation. Our data suggest that both peptides could initiate the differentiation process in all the studied cell lines. However, due to the different origins (HL-60 and K562—leukemic cell line *vs.* MEG-A2—megakaryoblastic origin) of the cell lines, the mechanism may differ. The increase in the *ELANE* mRNA expression noted in our experiments may also suggest enhancement of the migration of the tested cells. However, more research is needed to fully explain the mechanism of action of the VGVAPG and VVGPGA peptides in the HL-60, K562, and MEG-A2 cell lines.

**Highlights:**

• VGVAPG and VVGPGA peptides do not affect the metabolic activity of HL-60, K562, and MEG-A2 cells.

• mTOR and PPARγ proteins are involved in the mechanism of action of VGVAPG and VVGPGA peptides.

• Both peptides may initiate differentiation in HL-60, K562, and MEG-A2 cell lines.

**Graphical Abstract:**

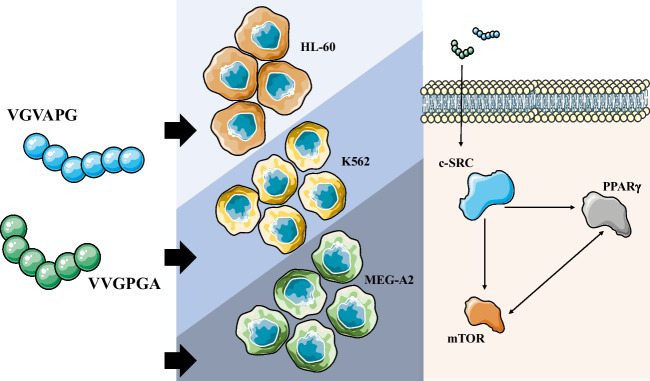

## Introduction

The extracellular matrix (ECM) is currently considered to be an important factor influencing the migration and progression of cancer cells [1]. Elastin is a major component of ECM, especially in organs and tissues which should be resilient and flexible, e.g., the aorta and large vascular vessels, lungs, skin, brain, and ligaments [2]. During aging and pathological conditions, elastin is degraded and elastin-derived peptides (EDPs) are created [3]. The most common repeated sequence identified in all EDPs is Val-Gly-Val-Ala-Pro-Gly (VGVAPG), which has the strongest affinity to the elastin binding protein (EBP) [4]. It has also been shown that this receptor transmits a signal to the cell and its effects are dependent on the EDP concentration, type of cells or tissues, and the time of exposure (reviewed in [5]). Moreover, some studies suggest that after EBP activation by EDPs, proto-oncogene tyrosine-protein (c-Src) kinase is involved in the signal transduction [6, 7]. c-SRC kinase transmits a signal of cell growth and proliferation, cell survival, and activation of cancer progression and invasion pathways, which seems to be crucial in cancer research [8–10].

It has been reported that the VGVAPG peptide exhibits chemotactic properties for bovine fibroblasts and human monocytes [11]. Moreover, this peptide is characterized by the strongest chemotactic properties in nanomolar (nM) concentrations, and cell migration decreases together with increasing peptide concentrations [11]. EDPs have been shown to attract the human mononuclear phagocyte (U937) cell line by chemotactic activity through activation of EBP, which may explain the inflammatory response in tissues in which the level of EDPs increases [12]. Similarly, soluble κ-elastin and EDPs bind with high affinity to EBP and, consequently, increase calcium mobilization and phosphatidylinositol breakdown involved in the signal transduction inside the cell, as shown inter alia by Varga et al. in primary polymorphonuclear leukocytes and monocytes isolated from human blood [13]. In neutrophils isolated from the blood of healthy subjects, the 1 µg/mL concentration of the VGVAPG peptide increased cell migration, while no significant changes in cell migration were observed in neutrophils obtained from patients with chronic obstructive pulmonary disease (COPD) (characterized by persistent inflammation) [14]. Moreover, in healthy subjects and those with stable COPD, the 1 µg/mL concentration of the VGVAPG peptide increased mRNA and protein expression of interleukin-6 (IL-6), tumor necrosis factor-alpha (TNFα), and interleukin-8 (IL-8), while a higher concentration (10 µg/mL) of the VGVAPG peptide did not significantly influence the above-described parameters [14]. Interestingly, both 1 and 10 µg/mL concentrations of the VGVAPG peptide increased reactive oxygen species (ROS) production in neutrophils obtained from healthy subjects and patients with COPD [13]. Summarizing, studies conducted to date show that it is crucial to test low physiological concentrations as well as high concentrations to eliminate false results. In addition to chemotaxis, available research also showed an effect of EDPs on other cells originating from the immune system and participating in differentiation and proliferation processes [13, 15, 16]. Exposure of mice to the VGVAPG peptide increased the number of CD4 + T cells, which are characterized by expression of interferon-gamma (IFN-γ) and interleukin-17A (IL-17A) in lungs, mediastinal lymph nodes (mLN), and spleen [15]. It has also been demonstrated that EDPs stimulate differentiation of human T helper type 1 (Th-1) lymphocytes [17].

Available data showed increased plasma elastase activity, and concentrations of EDPs were observed in patients with acute and chronic myeloid leukemia (CML) after hematopoietic stem cell transplantation [16]. Given the important role of elastin, EDPs, and ECM in cancer progression and metastasis and the lack of information on this topic, the study of the EDP mechanism of action in the case of leukemic cells seems to be a very important issue.

Therefore, the aim of our study was to investigate the mechanism of action of EDPs (VGVAPG and VVGPGA peptides) in cancerous cells derived from the immunological system, i.e., the HL-60, K562, and MEG-A2 cell lines. Moreover, an attempt to clarify the involvement of c-SRC kinase in the mechanism of action of EDPs was also undertaken.

## Materials and methods

### Reagents

Penicillin, streptomycin, dimethyl sulfoxide (DMSO), Tris–HCl, Tris-Base, bovine serum albumin (BSA), methanol, propan-2-ol, Bradford reagent, resazurin sodium salt, acrylamide/bisacrylamid, N,N,N′,N′-tetramethyl ethylenediamine (TEMED), ammonium persulfate (APS), and sodium-dodecyl sulfate (SDS) were purchased from Sigma–Aldrich (St. Louis, MO, USA). The VGVAPG and VVGPGA peptides were synthesized by LipoPharm.pl (Gdańsk, Poland). RPMI 1640 medium without phenol red was purchased from Corning (Manassas, USA). Fetal bovine serum (FBS), Universal RNA Purification Kit (E3598-02), Perfect Tricolor Protein Ladder™ (E3210-01), and Fast Probe qPCR Master Mix (2x) (E0422-03) were purchased from EURx (Gdańsk, Poland). The High Capacity cDNA Reverse Transcription Kit, as well as primers and TaqMan probes corresponding to a specific nucleotide sequence encoding *cMYC* (Hs00153408_m1), *KI67* (Hs04260396_g1), *PPARγ* (Hs00234592_m1), *GAPDH* (Hs02758991_g1), *NFKB2* (Hs01028901_g1), *ELANE* (Hs00236952_m1), and *MTOR* (Hs00234508_m1), and anti-mouse-HRP-conjugated (dilution 1:10 000, cat. 31,430) and anti-rabbit-HRP conjugated (dilution 1:10 000, cat. SH253595) secondary antibodies were obtained from Thermo Fisher Scientific (Foster City, CA, USA). The primary antibodies specific against human c-SRC (dilution 1:4000, cat. A19119), PCNA (dilution 1:2000, cat. A12427), mTOR (dilution 1:1000, cat. A11355), PPARγ (dilution 1:2000, cat. A11183), and ERK1/2 (dilution 1:2000, cat. A16686) proteins were purchased from ABClonal (Woburn, USA). The primary antibodies specific against human GAPDH (dilution 1:4000, cat. sc-47724), KI67 (dilution 1:400, cat. sc-23900), and PVDF membrane with 0.45-µm pore size were purchased from Santa Cruz Biotechnology (Santa Cruz, USA). The c-SRC inhibitor I was purchased from Cayman Chemical (Ann Arbor, USA).

Stock solutions of the test compounds were prepared in DMSO and were added to RPMI 1640 without phenol red. The final concentration of DMSO in the culture medium was always 0.1%.

### Cell culture and treatment

The human leukemia cell (HL-60) line and the human chronic myelogenous leukemia (K562) cell line were obtained from the American Type Culture Collection (ATCC distributor: LGC Standards, Łomianki, Poland). The human megakaryoblastic cell line (MEG-A2) was kindly gifted to our team by Professor Maciej Wnuk from the University of Rzeszów. The HL-60, K562, and MEG-A2 cells were maintained in RPMI 1640 medium without phenol red supplemented with 10% of FBS at 37 °C in a humidified atmosphere with 5% CO_2_. However, due to the performed proliferation-based analyses and physiological presence of some portion of EDPs in serum, all experiments were performed in a medium containing 1% FBS [18]. The cells were seeded in 96-well culture plates at a density of 5 × 10^3^ cells (for the resazurin reduction assay) per well and in T25 culture flask 1 × 10^6^ cells (for mRNA and Western blot) per flask. In this study, the representative of elastin-derived peptides (VGVAPG) and a synthetic elastin-like peptide (VVGPGA) were selected for the analyses.

### Resazurin reduction assay

The resazurin reduction cell viability and metabolism assay was conducted according to a modification of a previously described method [19]. Briefly, on the day of analysis, a working solution of 120 µM of resazurin sodium salt (2-time concentrated stock) was prepared in medium containing 1% of FBS. Subsequently, the cells were treated with increasing concentrations of VGVAPG or VVGPGA (1–100 nM and 1–100 µM) for 48 h and 72 h by adding 100 µL of a cell suspension with the tested concentration of the peptides and 100 µL of resazurin sodium salt stock (final volume 200 µL). Next, the plates were incubated for 48 h and 72 h at 37 °C and 5% CO_2_. After the specified periods, the fluorescence was measured with excitation and emission wavelengths of 530 and 590 nm, respectively, in a microplate reader—FilterMax F5 Multi-Mode (Molecular Devices, Corp., Sunnyvale, CA, USA), 4 h after the addition of the dye.

### Real-time PCR analysis of mRNAs specific to genes c*MYC*,* KI67*, *PPARγ*, *GAPDH*,* NFKB2*,* ELANE*, and *mTOR*

After the 24-h exposure to 10 nM of VGVAPG and 10 nM of VVGPGA peptides and in co-treatment with the c-SRC inhibitor I, samples of total RNA were extracted from the analyzed cell lines according to the manufacturer’s protocol. The RNA quality and quantity were determined spectrophotometrically at 260 nm and 280 nm (ND/1000 UV/Vis; Thermo Fisher NanoDrop, USA). Two-step real-time RT-PCR was conducted using the CFX Real Time System (BioRad, USA). The reverse transcription (RT) reaction was performed at a final volume of 20 µL with 800 ng of RNA (as a cDNA template) using the cDNA reverse transcription kit according to the manufacturer’s protocol. Products from the RT reaction were amplified using the FastStart Universal Probe Master kit with TaqMan probes as primers for specific genes encoding c*MYC*, *KI67*, *PPARγ*, *GAPDH*, *NFKB2*, *ELANE*, and *MTOR* according to the manufacturer’s protocol. Amplification was carried out in a total volume of 20 µL containing 1.0 µL of the RT product, and *GAPDH* was used as a reference gene.

### Cell number measurement

The TC20 automated cell counter (Bio-Rad Inc, Hercules, CA) was used to determine the number of HL-60, K562, and MEG-2A cells after the treatment with the tested compounds. Briefly, 1 × 10^5^ cells/well were grown in a 24-well plate in RPMI 1640 medium supplemented with 1% FBS and 10 nM of VGVPAG and 10 nM of VVGPGA or an equal volume of DMSO (control) for 24 or 48 h. After these time intervals, the cells were gently mixed, and 10 µL of the cell suspension was combined with 10 µL of trypan blue dye. Subsequently, the prepared cell solutions were analyzed in the TC20 cell counter (Bio-Rad). The result was presented as a percentage of the control.

### Western blot

The Western blot method was carried out as described by Skóra et al. without modifications [20]. Fifty micrograms of protein was used in SDS-PAGE electrophoresis. After enhanced chemiluminescence-based detection, the PVDF membranes were stripped with buffer containing 0.1% of SDS, 1.5% of glycin, and 1% of Tween-20 (pH = 2.2.). Subsequently, the membranes were blocked using 1% BSA in TBST, followed by adding other antibodies specific for a certain target or the reference protein. The GAPDH protein expression was used as a reference in each protein sample. The densitometry of the bands was measured using free GelQuantNet software (www.BiochemLabSolutions.com).

### Statistical analysis

The data are presented as means ± SD (standard deviation) of at least three independent experiments (*n* ⫺ 3). The normal distribution of the obtained results was confirmed using the Shapiro–Wilk test (*p* > 0.05)—all data met the aforementioned criteria. Subsequently, the data were analyzed by one-way analysis of variance (ANOVA) followed by Dunnett’s multiple comparison test (post-hoc) performed using GraphPad Prism 8.0 software with statistical mode. The statistical differences between the control and experimental groups were marked as follows: ∗ *p* < 0.05, ∗  ∗ *p* < 0.01, ∗  ∗  ∗ *p* < 0.001 versus control cells.

## Results

### Resazurin reduction measurement

**Fig. 1:**
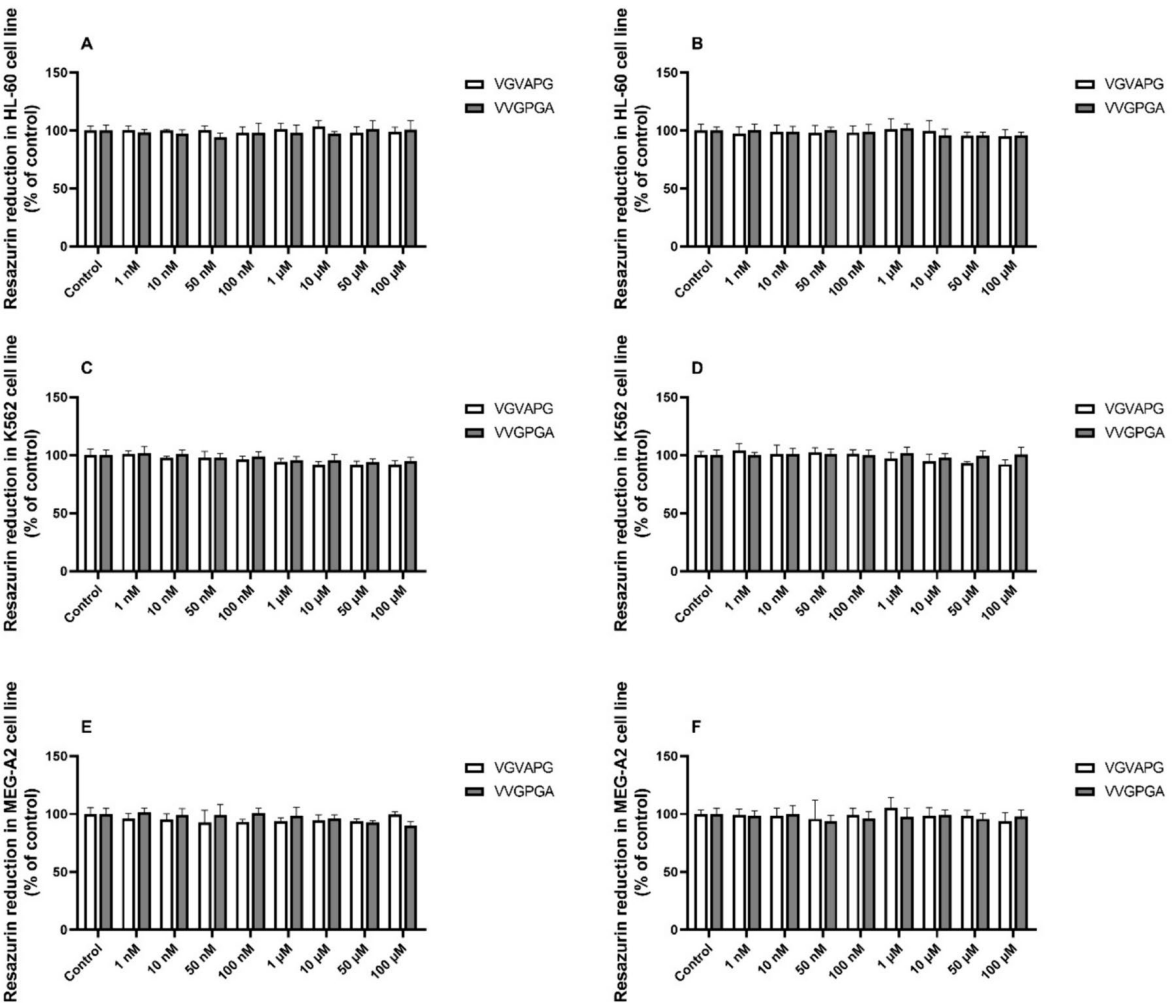
Cell metabolism. Resazurin reduction test after the 48-h (**A**, **C**, **E**) and 72-h (**B**, **D**, **F**) exposure of the HL-60 (**A**, **B**), K562 (**C**, **D**), and MEG-A2 (**E**, **F**) cell lines to increasing concentrations (1–100 nM and 1–100 µM) of the VGVAPG and VVGPGA peptides in RPMI 1640 medium supplemented with 1% FBS. The data are presented as means ± SD of three independent experiments. In each experiment, the treatment was repeated six times in the experimental group

In our experiments, after the 48-h and 72-h exposure, the studied peptides (VGVAPG and VVGPGA) in all studied concentrations (1–100 nM and 1–100 µM) did not significantly affect cell metabolism measured by the resazurin reduction test (Fig. 1).

### Gene expression analysis

**Fig. 2:**
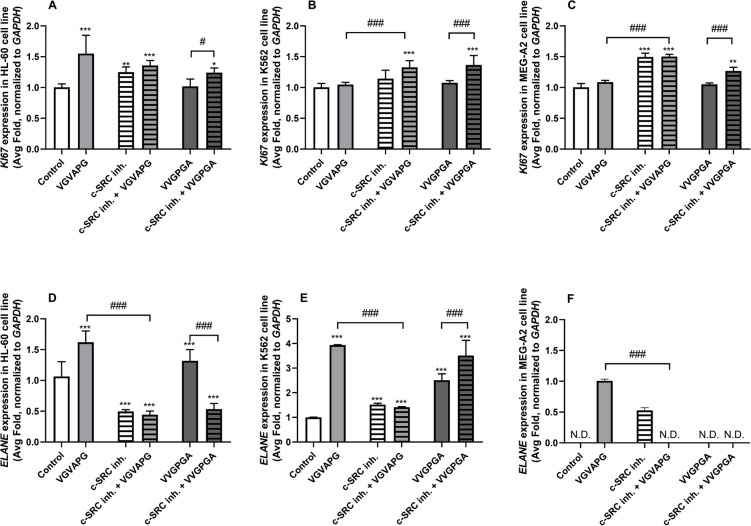
mRNA expression of *KI67* and *ELANE* genes in the leukemia cell lines. Effect of 10 nM VGVAPG and 10 nM VVGPGA on the mRNA expression of *KI67* (**A**–**C**) and *ELANE* (**D**–**F**) after the 24-h exposure of the HL-60 (**A**, **D**), K562 (**B**, **E**), and MEG-A2 (**C**, **F**) cell lines in RPMI 1640 medium supplemented with 1% FBS. The mRNA expression was normalized to the *GAPDH* gene. The data are expressed as means ± SD of three independent experiments, each of which consisted of six replicates per treatment group. *p < 0.05; **p < 0.01; ***p < 0.001 vs. the vehicle control. ###*p* < 0.001 vs. the respective peptide alone

In our experiments, after the 24-h exposure of the HL-60, K562, and MEG-A2 cell lines to the VGVAPG and VVGPGA peptides, only the VGVAPG peptide significantly increased the *KI67* mRNA expression (by 55.02%) compared to the control (Fig. 2A–C). In all the studied cell lines, the VVGPGA peptide co-treatment with the c-SRC inhibitor I increased the *KI67* mRNA expression, compared to the control (by 24.01% in the HL-60 cell line, by 36.62% in the K562 cell line, and by 26.99% in the MEG-A2 cell line) (Fig. 2A–C). Interestingly, the c-SRC inhibitor I used alone increased *KI67* mRNA only in HL-60 and MEG-A2 (by 24.95 and 49.54%, respectively, compared to the control).

In the HL-60 cell line, both VGVAPG and VVGPGA peptides increased *ELANE* mRNA by 62.02 and 31.87%, respectively, compared to the control (Fig. 2D). The c-SRC inhibitor I decreased the *ELANE* mRNA expression by 50.46%, compared to the control. The cell co-treatment with the c-SRC inhibitor and the VGVAPG or VVGPGA peptides decreased *ELANE* mRNA expression by 55.58 and 52.41%, respectively, compared to the use of VGVAPG or VVGPGA alone (Fig. 2D). In the K562 cell line, both VGVAPG and VVGPGA peptides increased *ELANE* mRNA by 293.64 and 151.09%, respectively, compared to the control (Fig. 2E). The c-SRC inhibitor I increased the *ELANE* mRNA expression by 51.78%, compared to the control. Interestingly, the cell co-treatment with the c-SRC inhibitor I and VGVAPG or VVGPGA peptides increased the *ELANE* mRNA expression by 41.37 and 250.94%, respectively, compared to the control (Fig. 2E).

In MEG-A2, *ELANE* mRNA expression was detected only in the group exposed to the VGVAPG peptide (which was taken as a reference value) and in the group treated with the c-SRC inhibitor I, which was 51.79% of the reference group (Fig. 2F).

**Fig. 3:**
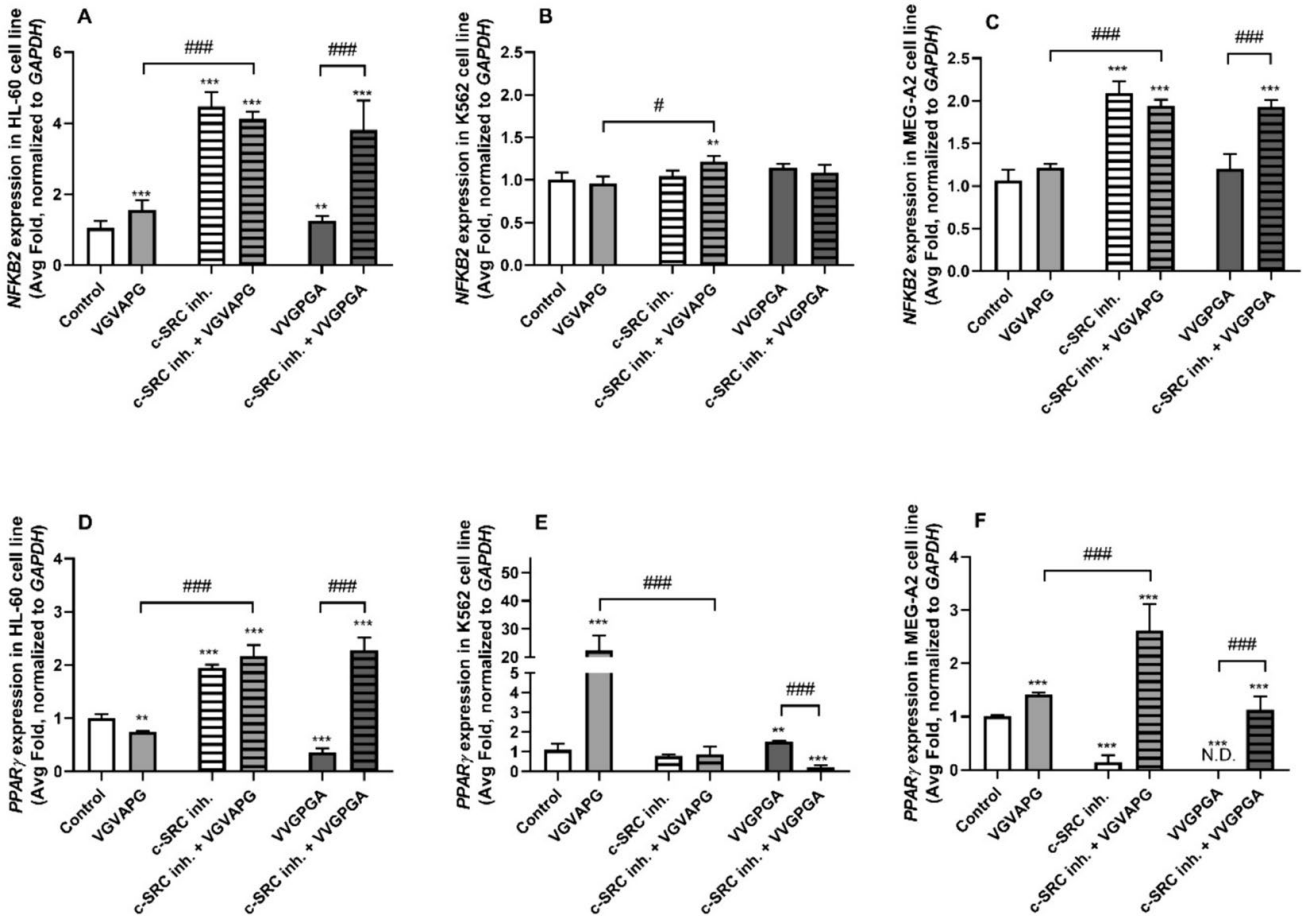
mRNA expression of *NFKB2* and *PPARγ* genes in the leukemia cell lines. Effect of 10 nM VGVAPG and 10 nM VVGPGA on the mRNA expression of *NFKB2* (**A**–**C**) and *PPARγ* (**D**–**F**) after the 24-h exposure of the HL-60 (**A**, **D**), K562 (**B**, **E**), and MEG-A2 (**C**, **F**) cell lines in RPMI 1640 medium supplemented with 1% FBS. The mRNA expression was normalized to the *GAPDH* gene. The data are expressed as means ± SD of three independent experiments, each of which consisted of six replicates per treatment group. *p < 0.05; **p < 0.01; ***p < 0.001 vs. the vehicle control. ###*p* < 0.001 vs. the respective peptide alone

In our experiments, the 24-h exposure of the HL-60 cell line to the VGVAPG and VVGPGA peptide increased the *NFKB2* mRNA expression by 56.00 and 25.82%, respectively, compared to the control (Fig. 3A). Interestingly, in the MEG-A2 cell lines, the VGVAPG and VVGPGA peptides did not induce statistically significant changes in the *NFKB2* mRNA expression, compared to the control (Fig. 3C). However, it should be noted that a slight but statistically insignificant increase in the *NFKB2* mRNA expression was observed. In both cell lines (HL-60 and MEG-A2), the c-SRC inhibitor I increased the *NFKB2* mRNA by 347.47 and 109.54%, respectively, compared to the control. In the HL-60 cell line, the co-treatment with the c-SRC inhibitor I and the VGVAPG or VVGPGA peptides increased the *NFKB2* mRNA expression by 311.69 and 281.41%, respectively, compared to the control (Fig. 3A). Similarly, the co-treatment with the c-SRC inhibitor I and the VGVAPG or VVGPGA peptides increased the *NFKB2* mRNA expression in the MEG-A2 cell line by 94.10 and 93.30%, respectively, compared to the control (Fig. 3C). In the case of the K562 cell line, a 21.79% increase in the *NFKB2* mRNA expression was observed only in the group co-treated with the c-SRC inhibitor I and the VGVAPG peptide, compared to the control (Fig. 3B).

After the 24-h exposure of the HL-60 cell line to the VGVAPG and VVGPAG peptides, *PPARγ* mRNA decreased by 25.28 and 63.72%, respectively, compared to the control. The c-SRC inhibitor I alone increased *PPARγ* mRNA by 94.87%, compared to the control. Interestingly, the cell co-treatment with the c-SRC inhibitor I and the VGVAPG or VVGPGA peptides induced a slight but statistically insignificant increase in *PPARγ* mRNA, compared to the treatment with the c-SRC inhibitor I alone (Fig. 3D). In the K562 cell line, the VGVAPG and VVGPGA peptides increased *PPARγ* mRNA by 2130.66 and 52.75%, respectively, compared to the control. The c-SRC inhibitor I did not change the *PPARγ* mRNA expression. Interestingly, no changes in the *PPARγ* mRNA expression were observed in the K562 cells co-treated with the c-SRC inhibitor I and the VGVAPG peptide, compared to the control. Similarly, a 77.20% decrease in the *PPARγ* mRNA expression was observed in the cells co-treated with the c-SRC inhibitor I and the VVGPGA peptide, compared to the control (Fig. 3E).

In the MEG-A2 cell line, VGVAPG increased the *PPARγ* mRNA expression by 41.56%, compared to the control. In turn, after the cell exposure to the VVGPGA peptide, no *PPARγ* mRNA expression was detected. The c-SRC inhibitor I alone decreased the *PPARγ* mRNA expression by 85.14%, compared to the control. Interestingly, the cell co-treatment with the c-SRC inhibitor I and the VGVAPG or VVGPGA peptides increased the *PPARγ* mRNA expression by 161.94 and 12.75%, respectively, compared to the control (Fig. 3F).

**Fig. 4:**
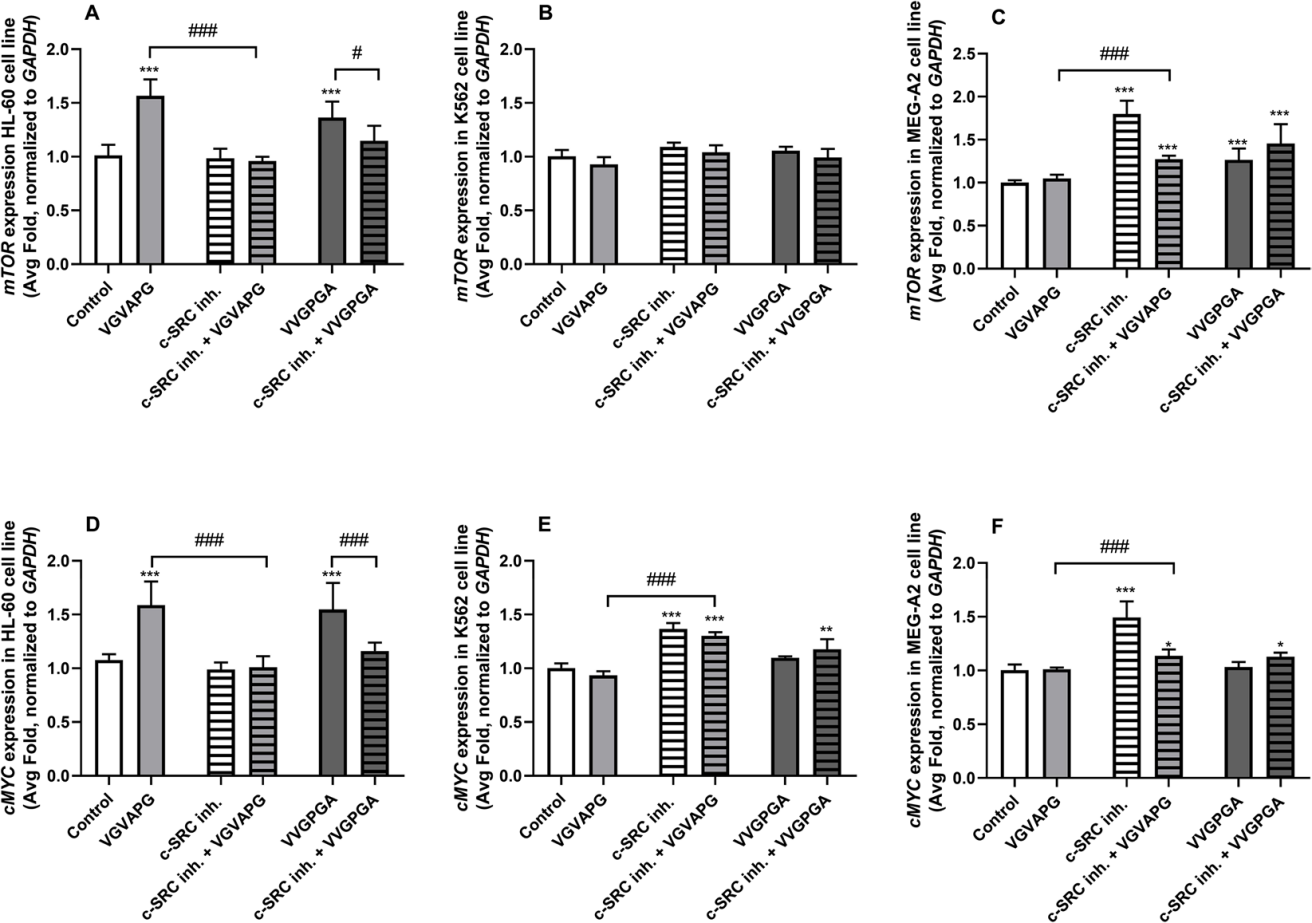
mRNA expression of *mTOR* and *cMYC* genes in the leukemia cell lines. Effect of 10 nM VGVAPG and 10 nM VVGPGA on the mRNA expression of *mTOR* (**A**–**C**) and *cMYC* (**D**–**F**) after the 24-h exposure of the HL-60 (**A**, **D**), K562 (**B**, **E**), and MEG-A2 (**C**, **F**) cell lines in RPMI 1640 medium supplemented with 1% FBS. The mRNA expression was normalized to the *GAPDH* gene. The data are expressed as means ± SD of three independent experiments, each of which consisted of six replicates per treatment group. *p < 0.05; **p < 0.01; ***p < 0.001 vs. the vehicle control. #*p* < 0.05, ###*p* < 0.001 vs. the respective peptide alone

After the 24-h treatment of the HL-60 cell line, both studied peptides (VGVAPG and VVGPGA) increased the *mTOR* mRNA expression by 56.50 and 36.44%, respectively, compared to the control. Interestingly, the cell exposure to the c-SRC inhibitor alone or the co-treatment with the c-SRC inhibitor I and the VGVAPG or VVGPGA peptides did not change the *mTOR* mRNA expression, compared to the control cells (Fig. 4A). In the K562 cell line, no significant changes in the *mTOR* mRNA expression were observed after the cell treatment with the VGVAPG or VVGPGA peptides alone or in the co-treatment with the c-SRC inhibitor I (Fig. 4B). In the case of the MEG-A2 cell line, the VVGPGA peptide and the c-SRC inhibitor I alone increased the *mTOR* mRNA expression by 26.48 and 79.96%, respectively, compared to the control. Interestingly, the cell co-treatment with the c-SRC inhibitor I and the VGVAPG peptide increased the *mTOR* mRNA expression by 27.28%, compared to the control. The cell co-treatment with the c-SRC inhibitor I and the VVGPGA peptide increased the *mTOR* mRNA expression by 45.59%, compared to the control (Fig. 4C).

After the 24-h treatment of the HL-60 cell line, both studied peptides (VGVAPG and VVGPGA) increased the *cMYC* mRNA expression by 58.76 and 54.71%, respectively, compared to the control. Interestingly, the cell exposure to the c-SRC inhibitor alone or the co-treatment with the c-SRC inhibitor I and the VGVAPG or VVGPGA peptides did not change the *cMYC* mRNA expression, compared to the control cells (Fig. 4D). In the K562 cell line, the c-SRC inhibitor I increased the *cMYC* mRNA expression by 36.63%, compared to the control. The K562 cell co-treatment with the c-SRC inhibitor I and the VGVAPG or VVGPGA peptides increased the *cMYC* mRNA expression by 30.27 and 17.82%, compared to the control (Fig. 4E). Finally, the VGVAPG and VVGPGA peptides did not change the *cMYC* mRNA expression in the MEG-A2 cell line, compared to the control. The c-SRC inhibitor I increased the *cMYC* mRNA expression by 49.45%, compared to the control. In the group co-treated with the c-SRC inhibitor I and VGVAPG or VVGPGA, a 13.60 and 12.83% increase in the *cMYC* mRNA expression was observed, respectively, compared to the control group (Fig. 4F).

### Cell number

**Fig. 5:**
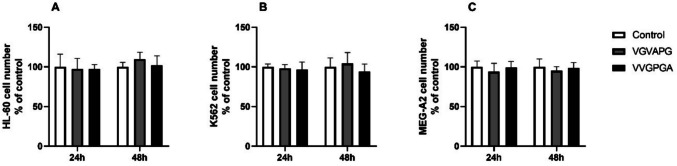
Cell number. Effect of 10 nM VGVAPG and 10 nM VVGPGA on the number of HL-60 (**A**), K562 (**B**), and MEG-A2 (**C**) cells in RPMI 1640 medium supplemented with 1% FBS. The data are expressed as means ± SD of three independent experiments and presented as a percent of the control

The experiments showed no statistically significant changes in the cell number after the 24-h exposure to the VGVAPG and VVGPGA peptides in all the studied cell lines (Fig. 5A–C). Similarly, after the 48-h exposure, both studied peptides did not cause statistically significant changes in the cell number in the HL-60, K562, and MEG-A2 cell lines (Fig. 5A–C).

### Protein expression measurement

#### HL-60 cell line

**Fig. 6:**
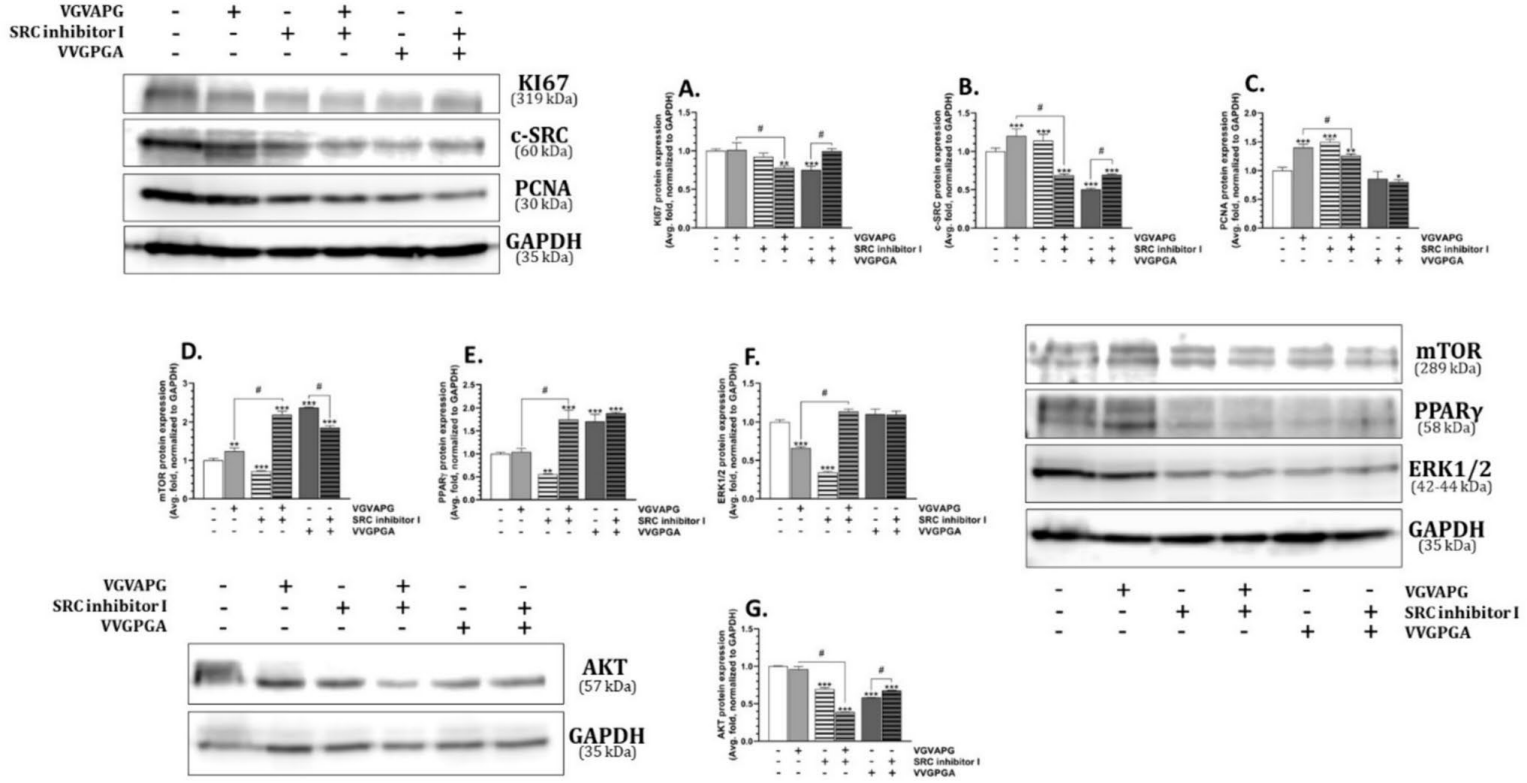
Protein expression in the HL-60 cell line. Relative protein expression of KI67, c-SRC kinase, PCNA, mTOR, PPARγ, and ERK1/2 after the 48-h treatment of the HL-60 cells with 10 nM VGVAPG, 10 nM VVGPGA, c-SRC kinase inhibitor I, and co-treatment with the studied peptides and inhibitor in RPMI 1640 medium supplemented with 1% FBS. The mean values ± SD with *, **, and *** are statistically different from the control group at *p* < 0.05, *p* < 0.01, and *p* < 0.001, respectively. Data denoted as # are statistically different between certain groups at *p* < 0.05

In the HL-60 cell line exposed to the VGVAPG peptide for 48 h, we did not observe any changes in the KI67 protein expression, while the VVGPGA peptide decreased the expression of this protein by 24.81%, compared to the control (Fig. 6A). Interestingly, the co-treatment of the HL-60 cell line with the c-SRC inhibitor I and the VGVAPG peptide decreased the KI67 protein expression, compared to the control. However, the cell co-treatment with the c-SRC inhibitor I and VVGPGA did not change the KI67 protein expression, compared to the control.

After the exposure of the HL-60 cells to the VGVAPG and VVGPGA peptides, the VGVAPG peptide increased while VVGPGA decreased the c-SRC kinase protein expression (increase by 20.08%, decrease by 49.52%), compared to the control (Fig. 6B). The c-SRC inhibitor I alone increased the c-SRC kinase protein expression by 14.10%, compared to the control. However, the cell co-treatment with the VGVAPG peptide and the c-SRC inhibitor I decreased the c-SRC kinase expression by 31.32%, compared to the control. In turn, the cell co-treatment with VVGPGA and the c-SRC inhibitor I induced a slight but statistically significant increase in the c-SRC kinase protein expression, compared to the treatment with the VVGPGA peptide alone (Fig. 6B).

The VGVAPG peptide increased the PCNA protein expression by 40.21%, while VVGPGA did not change PCNA protein expression, compared to the control (Fig. 6C). The c-SRC inhibitor I alone increased the PCAN protein expression by 49.61%, compared to the control. However, the cell co-treatment with the c-SRC inhibitor I and the VGVAPG peptide caused a slight but statistically significant 14.54% decrease in the PCNA protein expression, compared to the exposure to the VGVAPG peptide alone. Similarly, a slight but statistically insignificant decrease in the PCAN protein expression was observed in the cell co-treatment with the c-SRC inhibitor I and the VVGPGA peptide (Fig. 6C).

Both VGVAPG and VVGPGA peptides increased the mTOR protein expression by 24.52 and 137.74%, respectively, compared to the control (Fig. 6D). The c-SRC inhibitor I alone decreased the mTOR protein expression by 28.08%, compared to the control (Fig. 6D). Interestingly, the cell co-treatment with the VGVAPG peptide and the c-SRC inhibitor I increased the mTOR protein expression by 119.25%, compared to the control. In turn, the cell co-treatment with the VVGPGA peptide and the c-SRC inhibitor I decreased the mTOR protein expression, compared to the treatment with the VVGPGA peptide alone (Fig. 6D).

The VGVAPG peptide did not affect the PPARγ protein expression, but the VVGPGA peptide induced a 70.95% increase in the expression of this protein, compared to the control (Fig. 6E). The c-SRC inhibitor I decreased the PPARγ protein expression by 44.11%, compared to the control. In the cell co-treatment with the c-SRC inhibitor I and the VGVAPG or VVGPGA peptides, the PPARγ protein expression increased by 75.11 and 88.39%, respectively, compared to the control (Fig. 6E).

Both the VGVAPG peptide and the c-SRC inhibitor I decreased the ERK 1/2 kinase protein expression by 33.81 and 65.85%, respectively, compared to the control (Fig. 6F). Interestingly, the cell co-treatment with the c-SRC inhibitor I and the VGVAPG peptide did not change the ERK 1/2 kinase protein expression, compared to the control (Fig. 6F).

The exposure of the HL-60 cells to the c-SRC inhibitor I and the VVGPGA peptide decreased the AKT kinase protein expression by 30.49 and 41.61%, compared to the control (Fig. 5G). Similarly, the co-treatment with the c-SRC inhibitor I and the VGVAPG or VVGPGA peptides decreased the AKT kinase protein expression by 60.97 and 32.29%, respectively, compared to the control (Fig. 6G).

#### K562 cell line

**Fig. 7:**
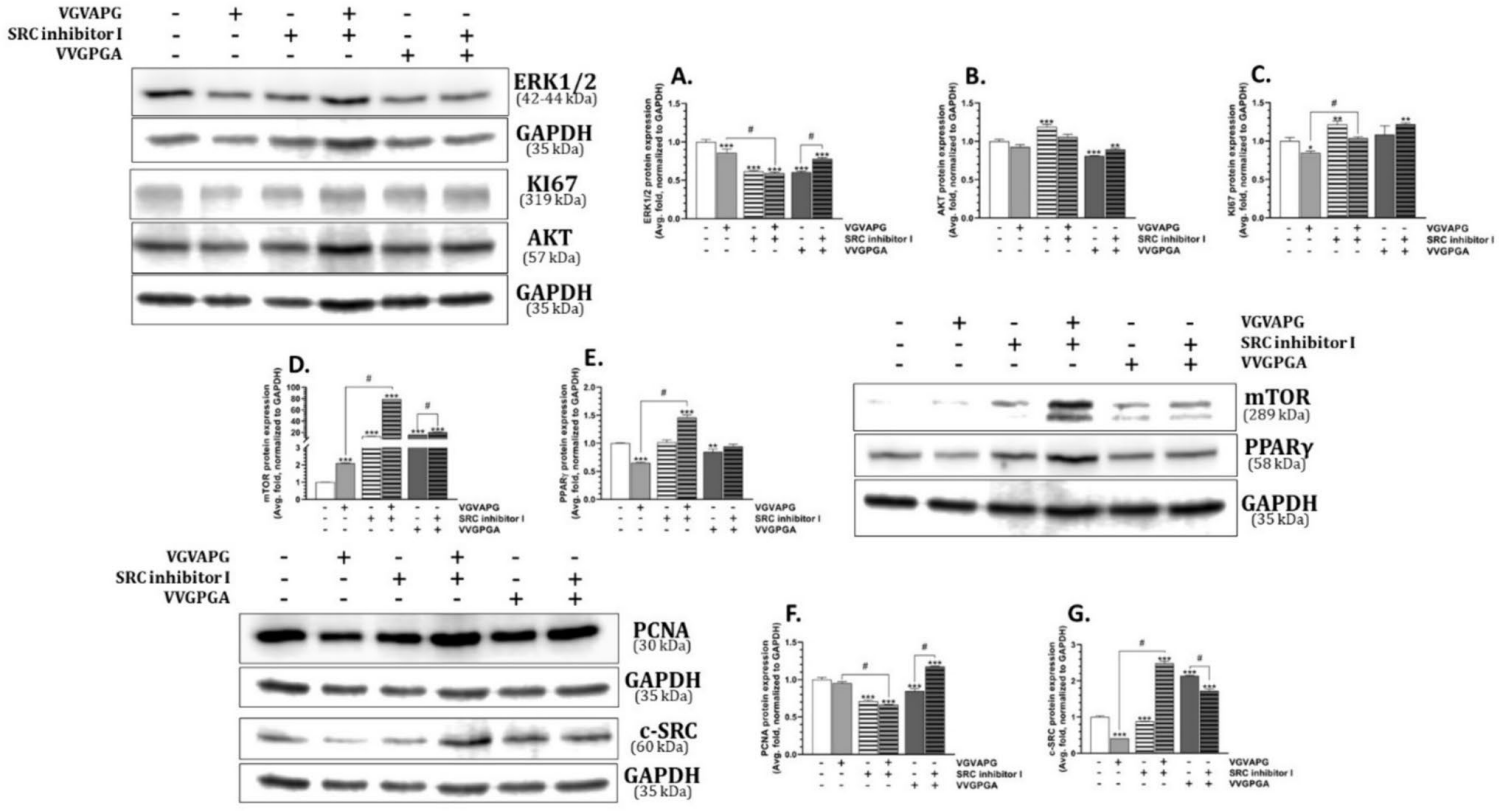
Protein expression in the K562 cell line. Relative protein expression of KI67, c-SRC kinase, PCNA, mTOR, PPARγ, and ERK1/2 after the 48-h treatment of the K562 cells with 10 nM VGVAPG, 10 nM VVGPGA, c-SRC kinase inhibitor I, and co-treatment with the studied peptides and inhibitor in RPMI 1640 medium supplemented with 1% FBS. The mean values ± SD with *, **, and *** are statistically different from the control group at *p* < 0.05, *p* < 0.01, and *p* < 0.001, respectively. Data denoted as # are statistically different between certain groups at *p* < 0.05

In the K562 cell line, the studied VGVAPG and VVGPGA peptides decreased the ERK 1/2 kinase protein expression by 14.35 and 39.50%, respectively, compared to the control (Fig. 7A). The c-SRC inhibitor I decreased the ERK 1/2 kinase protein expression by 38.33%, compared to the control (Fig. 7A). The co-treatment of the K562 cells with the c-SRC inhibitor I and the VGVAPG peptide did not change the ERK 1/2 kinase protein expression significantly, compared to the exposure to the c-SRC inhibitor I alone (Fig. 7A). Interestingly, the co-treatment of the K562 cells with the c-SRC inhibitor I and the VVGPGA peptide increased the ERK 1/2 kinase protein expression, compared to the treatment with the VVGPGA peptide alone (Fig. 7A).

After the 48-h exposure of the K562 cells to the VVGPGA peptide, an 18.98% decrease in the AKT kinase expression was observed, compared to the control (Fig. 7B). The c-SRC inhibitor I increased the AKT kinase expression by 18.79%, compared to the control. The cell co-treatment with the c-SRC inhibitor I and the VVGPGA peptide induced a slight but statistically insignificant increase in the AKT kinase protein expression (Fig. 7B). Interestingly, the VGVAPG peptide used alone and in the co-treatment with the c-SRC inhibitor I did not change the AKT kinase expression (Fig. 7B).

The VGVAPG peptide decreased the KI67 protein expression by 15.51%, compared to the control, while the c-SRC inhibitor I increased the expression of this protein by 21.51%, compared to the control (Fig. 7C). The cell co-treatment with the VGVAPG peptide and the c-SRC inhibitor I did not change the KI67 protein expression, compared to the control. Interestingly, the cell co-treatment with the VVGPGA peptide and the c-SCR inhibitor I increased the KI67 protein expression by 21.76%, compared to the control (Fig. 7C).

In the K562 cell line, both VGVAPG and VVGPGA peptides increased the mTOR protein expression by 109.62 and 1512.59%, respectively, compared to the control (Fig. 7D). Similarly, the c-SRC inhibitor I increased the mTOR protein expression by 1209.66%, compared to the control. The cell co-treatment with the c-SRC inhibitor I and the VGVAPG or VVGPGA peptides increased the mTOR protein expression compared to the treatment with the VGVAPG or VVGPGA peptides alone (increase by 7844.41 and 1967.48%, respectively) (Fig. 7D).

Both VGVAPG and VVGPGA peptides decreased the PPARγ protein expression by 34.86 and 15.09%, respectively, compared to the control (Fig. 7E). The c-SRC inhibitor I did not affect the PPARγ protein expression. The cell co-treatment with the c-SRC inhibitor I and the VGVAPG peptide increased the PPARγ protein expression by 45.83%, compared to the control (Fig. 7E).

After the 48-h exposure of the K562 cell line to the VGVAPG and VVGPGA peptides, only the VGVAPG peptide decreased the PCNA protein expression by 15.33%, compared to the control (Fig. 7F). The c-SRC inhibitor I decreased the PCNA protein expression by 28.91%, compared to the control. Moreover, in the group co-treated with the VGVAPG peptide and the c-SRC inhibitor I, a 33.58% decrease in the PCNA protein expression was observed, compared to the control (Fig. 7F). Interestingly, the cell co-treatment with the VVGPGA peptide and the c-SRC inhibitor I increased the PCNA protein expression by 17.74%, compared to the control (Fig. 7F).

In the present experiments, VGVAPG and the c-SRC inhibitor I decreased the c-SRC protein expression by 58.44 and 11.67%, respectively, compared to the control (Fig. 7G). Interestingly, the cell co-treatment with the VGVAPG peptide and the c-SRC inhibitor I increased the c-SRC protein expression by 148.87%, compared to the control. The VVGPGA peptide alone increased the c-SRC kinase expression by 114.19%, compared to the control (Fig. 7G). The cell co-treatment with the VVGPGA peptide and the c-SRC inhibitor I increased the c-SRC protein expression by 72.40%, compared to the control (Fig. 7G).

#### MEG-A2 cell line

**Fig. 8:**
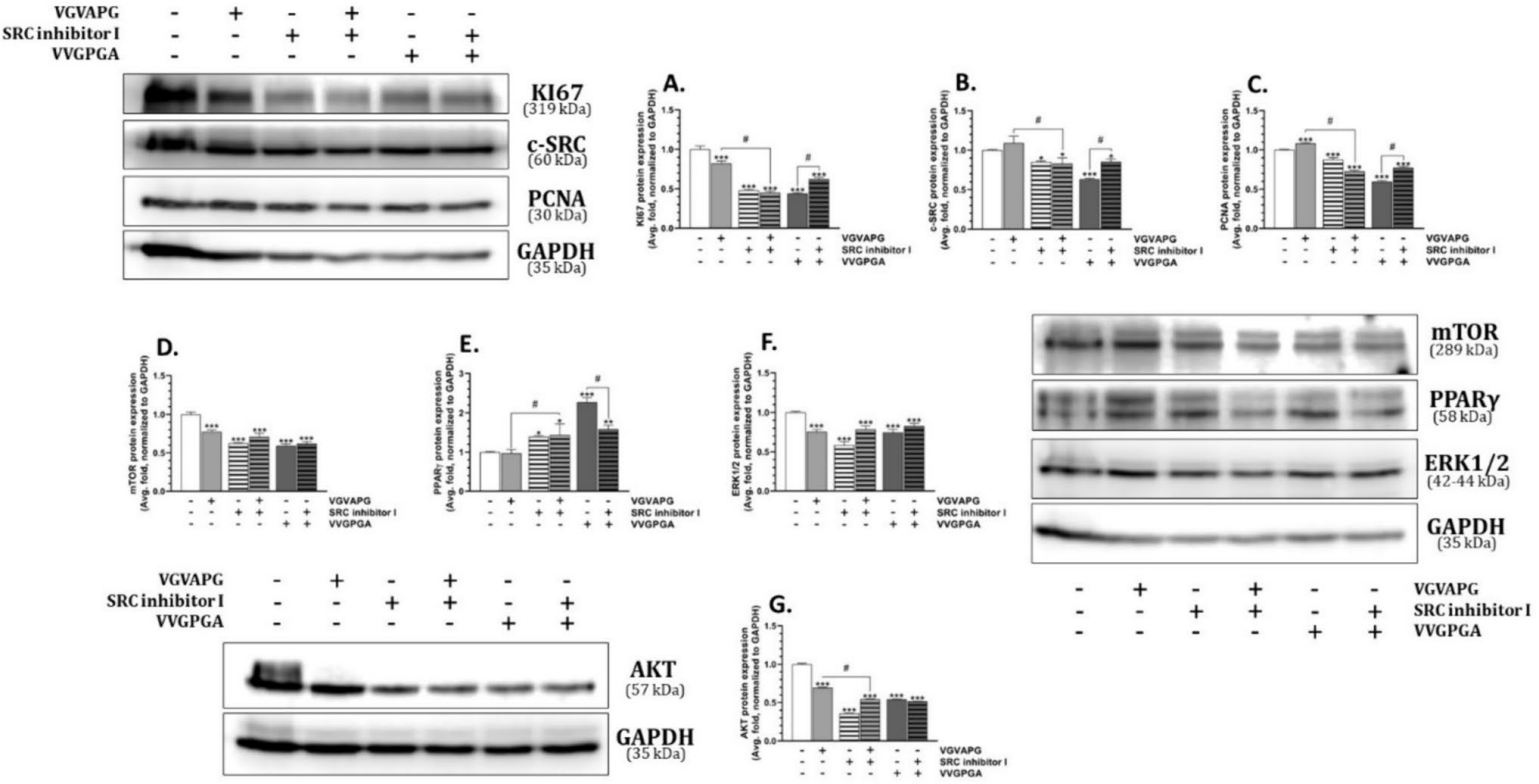
Protein expression in the MEG-A2 cell line. Relative protein expression of KI67, c-SRC kinase, PCNA, mTOR, PPARγ, and ERK1/2 after the 48-h treatment of the MEG-A2 cells with 10 nM VGVAPG, 10 nM VVGPGA, c-SRC kinase inhibitor I, and co-treatment with the studied peptides and inhibitor in RPMI 1640 medium supplemented with 1% FBS. The mean values ± SD with *, **, and *** are statistically different from the control group at *p* < 0.05, *p* < 0.01, and *p* < 0.001, respectively. Data denoted as # are statistically different between certain groups at *p* < 0.05

In the MEG-A2 cell line, the VGVAPG and VVGPGA peptides decreased the KI67 protein expression by 17.81 and 55.91%, respectively, compared to the control (Fig. 8A). The c-SRC inhibitor I alone and the c-SRC inhibitor I combined with the VGVAPG peptide decreased the KI67 protein expression by 51.92 and 54.79%, respectively, compared to the control (Fig. 8A). Interestingly, the cell co-treatment with the c-SRC inhibitor I and the VVGPGA peptide increased the KI67 protein expression by 37.81%, compared to the exposure to the VVGPGA peptide alone (Fig. 8A).

In the c-SRC inhibitor I-treated group and in the c-SRC inhibitor I with the VGVAPG peptide co-treatment variant, a 14.81 and 17.07% decrease in the c-SRC protein expression was observed, respectively, compared to the control (Fig. 8B). The VVGPGA peptide alone decreased the c-SRC protein expression by 36.82%, compared to the control. The cell co-treatment with the VVGPGA peptide and the c-SRC inhibitor I decreased the c-SRC protein expression by 14.39%, compared to the control (Fig. 8B).

The VGVAPG peptide increased the PCNA protein expression by 8.30%, compared to the control, while the VVGPGA peptide decreased the expression of this protein by 40.29% in the MEG-A2 cell line, compared to the control (Fig. 8C). The c-SRC inhibitor I used alone and combined with VGVAPG decreased the PCNA protein expression by 12.48 and 27.20%, respectively, compared to the control. A 22.60% decrease in the PCNA protein expression was observed in the cell co-treatment with the VVGPGA peptide and the c-SRC inhibitor I, compared to the control (Fig. 8C).

The VGVAPG and VVGPGA peptides and the c-SRC inhibitor I decreased the mTOR protein expression by 22.39, 40.78, and 37.47%, respectively, compared to the control (Fig. 8D). Similarly, the group co-treated with the c-SRC inhibitor I and the VGVAPG or VVGPGA peptides exhibited a 29.21 and 38.02% decrease in the mTOR protein expression, respectively, compared to the control (Fig. 8D).

The 48-h exposure to VGVAPG did not change the PPARγ protein expression significantly in the MEG-A2 cell line (Fig. 8E). The c-SRC inhibitor I alone and the VVGPGA peptide alone increased the PPARγ protein expression by 39.34 and 126.17%, respectively, compared to the control (Fig. 8E). In the group co-treated with the VGVAPG peptide and the c-SRC inhibitor I, a 43.86% increase in the PPARγ protein expression was observed, compared to the control (Fig. 8E). Interestingly, the cell co-treatment with the c-SRC inhibitor I and the VGVAPG peptide increased the PPARγ protein expression by 58.60%, compared to the control.

In the MEG-A2 cell line, the VGVAPG and VVGPGA peptides and the c-SRC inhibitor I decreased the ERK1/2 protein expression by 24.28, 25.40, and 41.50%, respectively, compared to the control (Fig. 8F). Similarly, in the group co-treated with the c-SRC inhibitor I and the VGVAPG or VVGPGA peptides, a 21.44 and 17.19% decrease in the ERK1/2 protein expression was observed, respectively, compared to the control (Fig. 8F).

After the 48-h exposure of the MEG-A2 cell line to the VGVAPG and VVGPGA peptides and the c-SRC inhibitor I, the AKT protein expression decreased by 30.36, 45.65, and 64.06%, respectively, compared to the control (Fig. 8G). Similarly, in the group co-treated with the c-SRC inhibitor I and the VGVAPG or VVGPGA peptides, a 45.62 and 47.97% decrease in the AKT protein expression was observed, respectively, compared to the control (Fig. 8G).

## Discussion

Our experiments showed that none of the tested concentrations (1–100 nM and 1–100 µM) of the VGVAPG and VVGPGA peptides changed the cell metabolism measured by the resazurin reduction test. Therefore, we can assume that the studied peptides are not toxic for the HL-60, K562, and MEG-A2 cell lines. Our experiments also showed that the studied peptides in all cell models mainly did not significantly increase the *KI67* mRNA and protein expression. Interestingly, the cell co-treatment with the studied peptides and the c-SRC inhibitor I significantly increased the *KI67* mRNA expression. At the protein level, the peptides decreased or did not affect the KI67 protein expression. However, the co-treatment of all the tested cells with the SRC inhibitor I and VGVAPG abolished or strengthened the effect of VGVAPG or VVGPGA by decreasing the KI67 protein expression, except for the K562 cells co-treated with the inhibitor and VVGPGA, which caused an increase in the KI67 protein expression. Given the well-described role of KI67 and PCNA proteins in cell cycle regulation, we can assume that the peptides do not stimulate proliferation of the HL-60, K562, and MEG-A2 cell lines. Our hypothesis is confirmed by the measurement of the cell number, which showed no significant changes in the proliferation of the studied cell lines after cell treatments with the tested peptides. The use of the c-SRC inhibitor I revealed that the VGVAPG and VVGPGA peptides may have different mechanisms of action. It is well-known that EDPs may act through different cellular receptors, with EBPs as the major receptors [21]. However, VGVAPG and VVGPGA peptides have different affinity for the EBP receptor, which results in differences between these peptides [22]. Therefore, it cannot be excluded that, after blocking the c-SRC kinase activated by EBP, the tested peptides activate a different molecular pathway. Indeed, previous studies revealed that EDPs increase the proliferation of lymphocytes; however, Péterszegi et al. and Szychowski et al. have also shown that κ-elastin can stimulate the proliferation or death of lymphocytes in a concentration-dependent manner as well as the amount of FBS used in the experiments [23–25].

Our theory may be confirmed by the level of c-SRC kinase protein expression after the stimulation with the VGVAPG and VVGPGA peptides. In the HL-60 and MEG-A2 cell lines, the VGVAPG peptide increased the c-SRC kinase protein expression, while the VVGPGA peptide decreased the expression of this protein. Interestingly, in the K562 cell line, we observed a reverse relationship, namely a decrease in the c-SRC kinase expression caused by the VGVAPG peptide, in contrast to VVGPGA, which was able to increase the expression of this protein. Interestingly, in all the cell lines, the c-SRC inhibitor I suppressed the changes induced by the tested peptides. c-Src kinase is involved in a number of processes such as regulation of embryonic development, cell growth and proliferation, cell survival, and activation of cancer progression and invasion pathways [8–10]. Moreover, a number of papers have confirmed that c-SRC kinase is crucial in signal transduction from EBP into the cell [21]. It has been confirmed that, through c-SRC kinase, EDPs activate Ca^2+^ ion influx, thus influencing neurosteroid production and secretion in mouse astrocytes [7, 26]. Interestingly, c-SRC kinase is upregulated in HL-60 and K562 cells during their differentiation [27, 28]. Moreover, we cannot exclude that the minor differences between the cell lines may result from their different origins and physiology.

As reported by Al Masri et al., c-SRC kinase regulates downstream proteins such as c-Myc (responsible for growth factor-induced mitogenesis), ERK kinases (which promote the G1/S phase), and AKT kinase, resulting in cell survival [29]. Additionally, c-SRC kinase can activate the mTOR signaling pathway through the AKT protein [30]. Therefore, in our study, we measured the mTOR, ERK 1/2, and AKT kinase protein expression as well as the *c-MYC* mRNA expression. Our data revealed that VGVAPG and VVGPGA peptides affected the AKT and ERK 1/2 kinase protein expression in a similar way in all the tested leukemia cell lines. It has been reported to date that a decrease in the total level of AKT and ERK 1/2 kinases is a result of proteasome-dependent degradation of these proteins, which may provide a counter-regulatory mechanism against overactivation of AKT and ERK 1/2 [31]. A decrease in the AKT and ERK1/2 protein level may suggest the inhibition of the cell cycle and proliferation and is consistent with the absence of cell proliferation observed in the study (confirmed by measuring the levels of KI67 and PCNA). In addition, besides regulation of cell proliferation and survival, AKT and ERK 1/2 kinases are able to affect cell metabolism, cell migration, and other cellular activities, in which the mammalian target of rapamycin kinase (mTOR) and peroxisome proliferator-activated receptor gamma (PPARγ) play a pivotal role [32]. Therefore, we decided to study the mTOR and PPARγ protein expression. Our experiments showed that both VGVAPG and VVGPGA peptides affected the mTOR and PPARγ protein expression in the HL-60, K562, and MEG-A2 cell lines in different ways.

Both peptides increased the mTOR protein expression, and the application of the c-SRC inhibitor potentiated the effect of the VGVAPG peptide in the HL-60 and K562 cell lines. In turn, in the MEG-A2 cell line, both peptides decreased the mTOR protein expression, and the c-SRC inhibitor did not influence changes caused by the tested peptides. The mTOR pathway plays an important role in the regulation of cell metabolism, survival, growth, and protein synthesis in both normal physiological and pathological conditions, especially in cancer [33]. To date, it has been shown that EDPs increase the mTOR protein expression in mouse choroidal endothelial cells (MCEC) [34]. An increase in the *mTOR* mRNA expression was also noted in a human undifferentiated neuroblastoma (SH-SY5Y) cell line after VGVAPG peptide stimulation [35]. Moreover, the involvement of the mTOR signaling pathway increases cell migration, MCEC tube formation, and angiogenesis. It has been reported that an increase in the mTOR protein expression in the HL-60 and K562 cell lines correlates with autophagy [36, 37]. In the case of the MEG-A2 cell line, it has been shown that the mTOR protein expression decreases during differentiation into megakaryocytes [38]. Similarly, a decrease in the mTOR protein expression in the MEG-01 cells was a result of cell differentiation and maturation [39]. To date, it has been well evidenced that mTOR activates the PPARγ signaling pathway [40].

In our experiments, the PPARγ protein expression pattern was similar in the HL-60 and MEG-A2 cell lines. More precisely, the VGVAPG peptide did not affect the PPARγ protein expression, while VVGPGA increased the expression of this protein. In the case of the K562 cell line, both peptides decreased the PPARγ protein expression. Interestingly, in all the studied cell lines, the c-SRC inhibitor I in the co-treatment with the VGVAPG peptide increased the PPARγ protein expression. Interestingly, the *PPARγ* mRNA expression in all the studied cell lines in the VGVAPG and VVGPGA peptide-treated groups was inversely correlated with the protein expression. This phenomenon has been well described and is mainly observed in the case of protein activation [41]. Moreover, it has been reported that the VGVAPG peptide activates PPARγ in mouse astrocytes and the SH-SY5Y cell line [35, 42, 43]. Therefore, we can assume that PPARγ is involved in the mechanism of action of the VGVAPG and VVGPGA peptides in all the studied cell lines. PPARγ is a multifunctional transcription factor with important regulatory roles in lipid metabolism, inflammation, cellular growth, differentiation, and apoptosis [44]. It is expressed in a variety of immune cells and in numerous leukemias and lymphomas [44]. Interestingly, a truncated alternative splicing variant of PPARγ (γ1tr) was discovered in the K562 cell line, in which it exhibited dominant negative activity [45]. This may explain the different expression of PPARγ from that in the other two cell lines studied. Therefore, the expression of c-SRC kinase and PPARγ in cells is different in the K562 cell line and in the other analyzed cell lines. Moreover, PPARγ activation can skew macrophage differentiation into a more anti-inflammatory phenotype [46]. To date, it has been demonstrated that the VGVAPG peptide decreased the level of interleukin-1 beta (IL-1β), which is a key pro-inflammatory cytokine, in mouse astrocytes in vitro [47]. On the other hand, different authors have consistently maintained that EDPs induce the production and/or secretion of IL-1α, IL-1β, and IL-6 in *ligamentum flavum* cells, synovial cells, and melanoma cell lines [48–50]. It should be noted that the effect of EDPs depends on the concentration, form of administration, exposure time, and tissue. Therefore, we believe that our data suggest that, through PPARγ activated by VGVAPG and VVGPGA peptides, the studied EDPs probably induce cell differentiation.

To date, it has been shown that the c-MYC protein inhibits erythroid differentiation in the K562 cell line, while the anti-c-MYC DNA oligomer induces granulocytic differentiation of human promyelocytic leukemia HL-60 cells in both serum-containing and serum-free media [51, 52]. On the other hand, c-MYC is essential for proper differentiation in megakaryocytes [51]. Therefore, we can assume that the low expression of *c-MYC* mRNA in HL-60 and the lack of changes in other cells promote differentiation. Moreover, differentiation of HL-60 and K562 cells requires nuclear factor kappa B (NF-κB) activation [53, 54]. Similarly, NF-κB is essential for the proper functioning of megakaryocytes and is also identified as a new target to dampen unwanted platelet activation [55]. Therefore, on the basis of our *NFKB2* gene expression results, we can assume that the studied peptides induce the differentiation process at least in the HL-60 cell line.

*ELANE* is a gene coding for human neutrophil elastase, a protease expressed early in neutrophil development, and has also been identified as an oncogene in various tumors associated with both cyclic and autosomal dominant neutropenia [56, 57]. Most likely, this may be a reason why the *ELANE* mRNA expression in the MEG-A2 cells was detected only in two groups, because these cells are already more differentiated than the HL-60 and K562 cell lines. It has been demonstrated that increased expression of neutrophil elastase protein and cDNA results in migration of HL-60 cells [58, 59]. Similarly, overexpression of neutrophil elastase promotes proliferation and inhibits apoptosis in K562 cells [60]. Unfortunately, in leukemia patients, *ELANE* is highly expressed and predicts poor survival [57]. Since the 1980s, it has been shown that EDPs initiate the migration of human monocytes in vitro [61]. Our observations suggest that an increase in the *ELANE* gene expression may facilitate cell migration.

## Conclusions

Our data show that the VGVAPG and VVGPGA peptides are not toxic to the studied cell lines. Moreover, given the role of the KI67 and PCNA proteins in the cell cycle and proliferation, it can be assumed that the peptides do not stimulate cell proliferation, suggesting potential initiation of the differentiation process in all the studied cell lines. However, given the different origins of the studied cell lines, the mechanism may be slightly different. The increase in the *ELANE* mRNA expression noted in our experiments may also suggest an increase in the migration of the tested cells. In summary, EDPs present in the human circulatory system may contribute to the spread of leukemic cells present in the body and/or their differentiation. Unfortunately, more research is needed to fully explain the mechanism of action of the VGVAPG and VVGPGA peptides in the HL-60, K562, and MEG-A2 cell lines.

## Data Availability

Data will be made available on request.

## Abbreviations

APS: Ammonium persulfate
BSA: Bovine serum albumin
CML: Chronic myeloid leukemia
COPD: Chronic obstructive pulmonary disease
COPD: Chronic obstructive pulmonary disease
c-Src: Proto-oncogene tyrosine-protein kinase
DMSO: Dimethyl sulfoxide
ECM: Extracellular matrix
EDPs: Elastin-derived peptides
FBS: Fetal bovine serum
IFN-γ: Interferon-gamma
IL-17A: Interleukin-17A
IL-1α: Interleukin 1 alpha
IL-1β: Interleukin 1 beta
IL-6: Interleukin 6
IL-8: Interleukin-8
MCEC: Mouse choroidal endothelial cells
mLN: Mediastinal lymph nodes
mTOR: Mammalian target of rapamycin kinase
NF-κB: Nuclear factor kappa B
PPARγ: Peroxisome proliferator-activated receptor gamma
ROS: Reactive oxygen species
SDS: Sodium-dodecyl sulfate
TEMED: N,N,N′,N′-Tetramethyl ethylenediamine
TNFα: Tumor necrosis factor-alpha
VGVAPG: Val-Gly-Val-Ala-Pro-Gly
VVGPGA: Val-Val-Gly-Pro-Gly-Ala
